# RETRACTION: LncRNA CCAT2 Promotes the Proliferation and Metastasis of Colorectal Cancer Through Activation of the ERK and Wnt Signaling Pathways by Regulating GNB2 Expression

**DOI:** 10.1002/cam4.70616

**Published:** 2025-02-07

## Abstract

**RETRACTION:** J. Tian, X. Cao, Z. Jiang, J. Wang, W. Fan, S. Zhang, S. Zhao, and J. Sun, “LncRNA CCAT2 Promotes the Proliferation and Metastasis of Colorectal Cancer Through Activation of the ERK and Wnt Signaling Pathways by Regulating GNB2 Expression,” *Cancer Medicine* 13, no. 17 (2024): e70169, https://doi.org/10.1002/cam4.70169.

The above article, published online on 03 September 2024, in Wiley Online Library (http://onlinelibrary.wiley.com/), has been retracted by agreement between the authors; the journal Editor‐in‐Chief, Stephen Tait; and John Wiley & Sons Ltd. The authors reported to the journal that a third party had detected multiple overlapping panels within and between Figures 2 and 7 in this article. The authors further stated that, upon reviewing the published article, they discovered multiple issues related to image overlap and redundancy. All parties agree that the overlapping and duplicated images constitute a major error in the article and it must be retracted.

**RETRACTION:** J. Tian, X. Cao, Z. Jiang, J. Wang, W. Fan, S. Zhang, S. Zhao, and J. Sun, “LncRNA CCAT2 Promotes the Proliferation and Metastasis of Colorectal Cancer Through Activation of the ERK and Wnt Signaling Pathways by Regulating GNB2 Expression,” *Cancer Medicine* 13, no. 17 (2024): e70169, https://doi.org/10.1002/cam4.70169.

The above article, published online on 03 September 2024, in Wiley Online Library (http://onlinelibrary.wiley.com/), has been retracted by agreement between the authors; the journal Editor‐in‐Chief, Stephen Tait; and John Wiley & Sons Ltd. The authors reported to the journal that a third party had detected multiple overlapping panels within and between Figures 2 and 7 in this article. The authors further stated that, upon reviewing the published article, they discovered multiple issues related to image overlap and redundancy. All parties agree that the overlapping and duplicated images constitute a major error in the article and it must be retracted.

